# Prévalence et facteurs de risque du surpoids et de l'obésité dans une population d'enfants scolarisés en milieu urbain à Sfax, Tunisie

**DOI:** 10.11604/pamj.2014.17.57.3351

**Published:** 2014-01-25

**Authors:** Sofien Regaieg, Nadia Charfi, Lobna Trabelsi, Mahdi Kamoun, Habib Feki, Sourour Yaich, Mohamed Abid

**Affiliations:** 1 Unité de Recherche Obésité-Syndrome Métabolique, Service d’‘Endocrinologie, CHU Hédi Chaker, Sfax, Tunisie; 2 Service Médecine Communautaire et d'Epidémiologie. CHU Hédi Chaker, Sfax, Tunisie

**Keywords:** Enfant, obésité, surpoids, facteurs de risque, milieu scolaire, child, obesity, overweight, risk factors, school

## Abstract

**Introduction:**

L'objectif de ce travail etait d’étudier la prévalence du surpoids et de l'obésité chez un groupe d'enfants d’âge scolaire, habitant la ville de Sfax en Tunisie, et identifier les facteurs favorisant la prise pondérale

**Méthodes:**

Il s'agossait d'une enquête descriptive transversale était réalisée en 2011 sur un échantillon représentatif d’élèves recrutés dans 11 écoles primaires publiques. Des informations concernant les caractéristiques sociodémographiques, les habitudes alimentaires et le comportement sédentaire pour chaque élève ont été précisées au moyen d'un questionnaire

**Résultats:**

Nous avons colligé 1529 élèves, âgés entre 9 et 12 ans et se répartissant en 787 garçons (51,14%) et 747 filles (48,86%). Selon les seuils de référence de l'IOTF, la fréquence de l'obésité était de 2,4% et celle du surpoids était de 6,3%. L'obésité était significativement associée à l'obésité parentale, un niveau socioéconomique élevé, la prise de plus de deux goûters par jour et à l'activité sédentaire.

**Conclusion:**

L'identification des facteurs de risque du surpoids et de l'obésité infantile permettrait de dépister les enfants à risques afin de leur proposer des mesures de prévention adaptées. Ces mesures de prévention devraient inclure non seulement des approches individuelles, mais aussi l'environnement social et physique de l'enfant.

## Introduction

La fréquence du surpoids et de l'obésité augmente de façon très rapide notamment chez les enfants, devenant ainsi un problème majeur de santé publique à l’échelle mondial [1, 2]. La Tunisie ne semble pas être épargnée par le phénomène d′obésité infantile dont la prévalence est en augmentation alarmante [3–6]. L'obésité infantile constitue un facteur de risque majeur de maladies cardiovasculaires, et peut entrainer des problèmes articulaires, respiratoires, métaboliques, endocriniens ou même encore orthopédiques. Au delà des conséquences somatiques, l'obésité infantile peut entraîner de nombreux troubles psychosociaux. L'obésité chez les enfants présente en outre un risque important de persistance à l’âge adulte. Ces complications multiples de l'obésité soulignent l'intérêt d'une approche préventive efficace qui devrait être instaurée dès l'enfance [7]. Le but de ce travail est d’étudier la prévalence et les facteurs de risque associés au surpoids et à l'obésité chez des adolescents scolarisés en vue d'orienter la mise en place d'un programme de prévention.

## Méthodes

**Type de l’étude:** Il s'agit d'une enquête descriptive, transversale qui a porté sur un échantillon représentatif d’élèves durant une période de 10 mois allant du mois de Novembre 2010 jusqu'au mois d'Avril 2011.

**Population étudiée:** La population d’étude était composée de 1529 enfants âgés de 9 à 12 ans, scolarisés dans 11 écoles primaires publiques de la région de Sfax. Ces enfants étaient sélectionnés au hasard parmi des élèves inscrits dans des classes 3éme, 4éme et 5éme année primaire.

### Recueil des données

#### Description du protocol

**Mesure des paramètres anthropométriques:** Le poids (en kg) était mesuré avec une précision proche de 50 grammes entre 8h du matin et midi chez des sujets déchaussés en tenue légère et avec vessie vide. La taille (en mètre) était mesurée avec une précision de 0,5 cm sur des sujets déchaussés, pieds joints bien à plat sur le sol, dos, fesses et talons étaient appliqués contre la planche verticale de la toise et la tête placée en position horizontale de sorte que la ligne de vision soit perpendiculaire au corps. L′indice de masse corporelle (IMC) était calculé en divisant le poids par la taille au carré IMC = Poids/Taille^2^ (kg/m^2^).

**Evaluation du statut pondéral:** Pour évaluer le statut pondéral des élèves, nous avons utilisé:

Les données définies par l'International Obesity Task Force (IOTF). La courbe de centile passant par un IMC égal à 25 à l’âge de 18 ans permet de définir le seuil du surpoids, obésité incluse (IOTF 25) et la courbe des centiles passant par un IMC égal à 30 à l’âge de 18 ans permet de définir le seuil de l'obésité (IOTF 30) [8].

Les données de références françaises qui définissent le poids normal par une IMC 90ème percentile, le surpoids par une IMC comprise entre 90ème et 97ème percentile et l'obésité par une IMC 97ème percentile, en tenant compte de l’âge et du sexe [9].

**Questionnaire:** Les élevés participants avaient reçu un questionnaire à remplir en collaboration avec leurs parents. Ces derniers étaient préalablement informés de la réalisation de l′étude, de ses objectifs et de ses modalités. Toutes les données étaient recueillies dans le respect de la confidentialité et de l′anonymat. Les informations recueillies à partir de ce questionnaire concernaient certains facteurs de risque de l'obésité: poids à la naissance, corpulence parentale, niveau socio-économique (métiers des parents, conditions de logement, moyens de déplacement), les habitudes alimentaires (prise de différents repas de la journée, grignotage et nombre de goûters par jour), activité physique (pratique de l′activités physique en dehors de l′école et le moyen de transport utilisé pour aller à l′école) et la sédentarité (nombre d′heures passées devant la télévision, les jeux vidéo et l′ordinateur pour un jour d′école et un jour de repos). Nous avons, exclu de cette partie les élèves qui n′ont pas rendu le questionnaires ou pour lesquels les questionnaires étaient « incomplets ».

**Analyse statistique:** La saisie des données était réalisée en utilisant le logiciel SPSS dans sa 18ème version. L′analyse des données comporte deux volets: une analyse uni-variée, pour rechercher des corrélations éventuelles entre le surpoids (obésité incluse) et les différentes variables associées; une analyse multi-variée, par régression logistique, afin de dégager les facteurs les plus discriminants associés au surpoids dans notre population.

L'analyse des données a fait appel au test de chi-deux (χ2) pour la comparaison des fréquences et à la régression logistique pour l′analyse multi-variée. Les résultats du risque associés à l′obésité étaient exprimés par les Odds Ratio (OR) ajustés avec leurs intervalles de confiance à 95%. Le seuil de significativité statistique était fixé à 5%.

## Résultats

Notre population comportait 1529 enfants dont 782 garçons (51,4%) et 747 filles (48,86%) d′âge moyen 10,12 ± 0,79 ans. Les enfants avaient tous eu une mesure du poids et de la taille et un calcul de lIMC. Le poids moyen des enfants participants était de 31,8 ± 7,36 kg. L′IMC moyen était de 16,72 ± 2,57 kg/m^2^ (extrêmes: 11,2 et 31,5 kg/m^2^). Tableau 1) illustre la répartition des élèves examinés selon le poids, la taille et l'IMC.

**Tableau 1:** Caractéristiques anthropométriques de la population d’étude

	Minimum	Maximum	Moyenne	Ecart- type
Poids (kg)	19	71	31,8	7,36
Taille (cm)	114	164	137,2	8,22
IMC (kg/m^2^)	11,2	31,5	16,7	2,57

### Prévalence du surpoids et d′obésité

**Figure 1:**
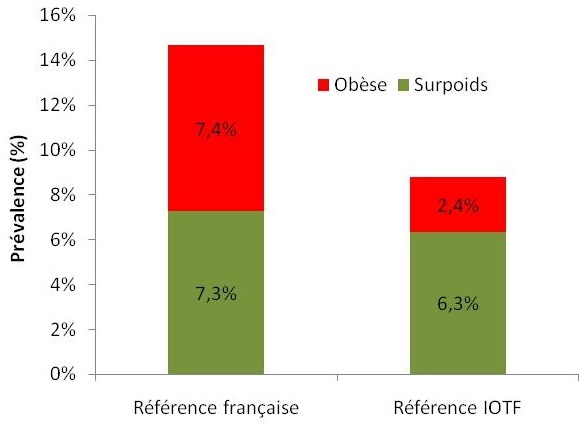
Prévalence de l'obésité et du surpoids selon les courbes de référence Française et les seuils de l'International Obesity Task Force de la population d’étude épidémiologique

Figure 1 montre la fréquence de la surcharge pondérale et de l'obésité selon l'ITOF et les courbes françaises. Selon les définitions de l'IOTF, la fréquence de la surcharge pondérale était de 6,3% et celle de l′obésité était de 2,4%, alors que ces fréquences étaient de 7,3% et 7,4% respectivement selon les références Françaises.Tableau 2 illustre la répartition de la corpulence par sexe selon l'ITOF et les courbes françaises. En se référant aux courbes françaises, la prévalence de la surcharge pondérale était significativement plus élevée chez les filles (9,1% vs 5,5%; P < 0,05). Selon l'ITOF, il n'y avait pas de différence significative pour la prévalence de l'obésité entre les deux sexes. La répartition des élèves selon leur statut pondéral par tranche d′âge (Tableau 3) avait montré que la fréquence d′obésité était plus importante dans le groupe de tranche d’âge entre 11 et 12 ans sans que la différence soit statistiquement significative.

**Tableau 2:** Prévalence de l'obésité et du surpoids selon le sexe

Référence	Prévalence	Filles	Garçons	P
**IOTF**	Surpoids	6,8%	6,10%	NS
	Obésité	2%	2,8%	NS
**Française**	Surpoids	9,1%	5,5%	P < 0,05
	Obésité	7,2%	7,5%	NS

IOTF: International Obesity Task Force; NS: non significatif

**Tableau 3:** Prévalence de l'obésité et du surpoids selon l’âge

Référence	Prévalence	9-10 ans	10-11ans	11-12ans	p
**IOTF**	Surpoids	6,6%	6,10%	6,3%	NS
	Obésité	2%	3%	2,4%	
**Française**	Surpoids	6,8%	7,7%	7,10%	
	Obésité	6,5%	7,2%	8,2%	

IOTF: International Obesity Task Force

### Facteurs de risque associés au surpoids (obésité incluse; référence IOTF)

Sur les 1526 enfants pour les quels des mesures anthropométriques ont été prises, 1262 élèves ont rendu les questionnaires complets sans données manquantes. 108 enfants étaient en surpoids et 1158 en poids normal. Le sex-ratio filles/garçons était de 0,95. Parmi les élèves recrutés, 6,7% avaient un poids à la naissance moins de 2,5 kg, 86,7% avaient un poids entre 2,5 et 4 kg et 6,6% avaient un poids supérieur à 4 kg. La fréquence du surpoids (obésité incluse) dans ces trois groupes était respectivement 9,4%, 8,2% et 12%. Aussi, nous n′avons pas constaté de variation significative entre les enfants qui avaient un poids de naissance inférieur à 2,5 kg ou supérieur à 4kg et les autres (OR= 1,34: IC à 95%: (0.79-2.29) p = 0,28), Par contre, plusieurs autres facteurs étaient significativement liés au surpoids (obésité incluse) (Tableau 4). C'est ainsi que l′obésité parentale était positivement corrélé au risque d′obésité infantile (OR= 2,62: IC à 95%: (1,44-4,78) p < 0,001), Le haut niveau socio-économique avait également émergé comme facteur favorisant le surpoids et l′obésité infantile. Cest ainsi que la fréquence du surpoids était significativement plus élevée chez les enfants qui habitaient une Villa (OR =1,83: IC à 95%: (1,23-‘2,27) p = 0,003) et chez les enfants dont les parents possédaient une voiture (OR =1,92: IC à 95%:(1,29-2,87) p = 0,001).

**Tableau 4:** Résultats de l'analyse uni-variée et multi-variée: Odds ratio (OR) et intervalles de confiance à 95% (IC)

Variables		Effectif (n)	Univariée OR (CI 95%)	p	Multivariée OR ajusté (CI 95%)	p
Un des parents au moins avait un problème de surpoids	Oui	82	2,62 (1,44 - 4,78)	0,001		
	Non	1184				
Poids de naissance inférieur à2,5kg ou supérieur à 4kg	Oui	168	1.34(0.79-2.29)	0,28		
	Non	1098				
Père était un ouvrier ou un employer	Oui	829	0,78 (0,52-1,17)	0,23		
	Non	437				
Le logement était une Villa	Oui	678	1,83 (1,23-2,27)	0,003		
	Non	588				
Parent possède une voiture	Oui	551	1,92 (1,29-2,87)	0,001		
	Non	715				
Dépasse parfois le petit déjeuner	Oui	556	1,02 (0,81-1,79)	0,35		
	Non	710				
Habituellement mange entre les repas	Oui	398	1,31 (0,87-1,98)	0,19		
	Non	868				
Habituellement prend plus de deux goûters par jour	Oui	85	2,74 (1,53-4,92)	0,001	4,25 (1,43-12,60)	0,009
	Non	1181				
Se rend à l’école en vélo ou à pied	Oui	1177	0,57 (0,3-1,08)	0,083		
	Non	89				
Pratique de l'activité physique dans un club	Oui	108	0,81 (0,42-1,55)	0,52		
	Non	1158				
Regarde la télé plus de deux heures pendant les jours d’école	Oui	246	1,2 (0,80-2,04)	0,31		
	Non	1020				
Regarde la télé plus de 4heures pendant les jours de repos	Oui	812	4,9 (2,68- 9,1)	< 0,001	4,39 (1,57-12,24)	0,005
	Non	454				
Joue aux jeux vidéo ou rester devant l'ordinateur plus de deux heures pendant les jours de repos (concernant les élèves qui possèdent un ordinateur ou de jeux vidéo)	Oui	142	2,58 (1,24-5,37)	0,009	2,38 (1,02-5,57)	0,046
	Non	86				
Joue aux jeux vidéo ou rester devant l'ordinateur plus d'une heure pendant les jours d’école (concernant les élèves qui possèdent un ordinateur ou de jeux vidéo)	Oui	38	3,34 (1,58-7,01)	0,001	2,39 (1,01-5,63)	0,047
	Non	190				

L'analyse des habitudes alimentaires nous a permis de déduire que le fait de prendre plus de deux goûters par jour était significativement associé au surpoids (OR= 2,74: IC à 95%: (1,53-4,92) p < 0,001). Par contre, la présence de troubles du comportement alimentaire à type grignotage et le fait de dépasser le petit déjeuner n'affectaient pas significativement la prévalence du surpoids avec simultanément (p= 0,19) et (p= 0,35). La façon dont les enfants se rendaient à l’école c′est-à-dire à pieds ou en vélo versus en voiture n'avait pas d'influence sur le poids (p = 0,083). Similairement, il n'avait pas de différence significative entre les 2 groupes d'enfants (surpoids vs corpulence normale) en ce qui concerne la pratique ou non d'activités physiques dans un club sportif (p =0,52). La prévalence de la surcharge pondérale et de l'obésité étaient significativement plus élevée chez les élèves qui regardaient la télévision plus que 4 heures par jour pendant les jours de repos (OR =4,9: IC à 95%: (2,68-9,1) p < 0,001). Cependant, elle n′était pas significative pour ceux qui regardent la télé plus de deux heures pendant les jours d′école (p = 0,31).

Enfin, le temps consacré aux jeux vidéo et le temps passé devant l'ordinateur influence significativement le poids des enfants puisque la fréquence du surpoids était plus élevée parmi ceux qui jouaient aux jeux vidéo ou rester devant l′ordinateur plus que 2 heures pendant les jours de repos (OR= 2,58: IC à 95%: (1,24-5,37) p = 0,009) et une heure par jour les jours de classe (OR= 3,34: IC à 95%: (1,58-7,01) p = 0,001).

Après une analyse multi-variée, parmi les sept facteurs associés significativement au surpoids, seuls quatre étaient des facteurs indépendants (Tableau 4). En effet, la fréquence de surpoids était plus élevée chez les enfants qui prenaient plus de deux goûters par jours (p = 0,009), les enfants qui regardaient la télévision plus de quatre heures pendant les jours de repos (p = 0,005) et les enfants qui jouaient aux jeux vidéo ou rester devant l′ordinateur plus d′une heure pendant les jours d′école et deux heures pendant les jours de repos (p < 0,05).

## Discussion

L'ensemble des études menées à la fois dans les pays industrialisés et en développement indiquent une augmentation rapide du nombre d'enfants ayant un surpoids ou une obésité. En 2010, selon les standards de l'OMS, 43 millions d'enfants (dont 35 millions dans les pays en voie de développement) étaient considérés comme étant en surpoids ou obèses; 92 millions étant à risque de surpoids. La prévalence du surpoids (obésité incluse) de l'enfant est passée de 4,2% en 1990 à 6,7% en 2010. Cette tendance devrait atteindre 9,1% en 2020, représentant approximativement 60 millions d'enfants. Le taux d'obésité dans les pays développés étant 2 fois plus élevé que celui des pays en voie de développement [1]. En France, la prévalence de l'obésité infantile était de 10 à 12%. Elle avait plus que doublé depuis les années 1980 [3]. L'augmentation de l'obésité sévère étant plus rapide que celle de l'obésité modérée [3]. Aux Etats-Unis, 25% des enfants et adolescents présentent un IMC situé entre le 90ème et le 97ème percentile, et 11% sont considérés comme obèses, ce qui correspond à un IMC au-delà du 97ème percentile [10]. La Tunisie n’échappe pas à ce phénomène épidémique puisque les enquêtes réalisées à l’échelle nationale avaient bien montré que la prévalence de l'obésité chez l'enfant ne cesse d'augmenter [4–6]. Dans une étude récente menée par Boukthir et al. et incluant des écoliers tunisiens âgés de 6 à 12 ans, la prévalence du surpoids était de 19,7% et celle de l'obésité de 5,7% selon l'IOTF [11]. Dans notre étude, la prévalence du surpoids (obésité incluse) était de 8,7%. La fréquence de la surcharge pondérale était de 6,3% et celle de l′obésité était de 2,4% selon l'ITOF. Selon les courbes de référence Française, ces deux fréquences étaient de 7,3% et 7,4% respectivement. Certaines études [3, 12, 13] mais pas toutes [3], avaient constaté que le surpoids est plus important chez les filles. Dans notre série la prévalence de l'obésité était comparable entre les deux sexes. Cependant, et selon les références françaises, il existait une différence significative entre les deux sexes concernant la surcharge pondérale (9,2% des filles et 5,5% des garçons, p < 0,05). La prévalence de l'obésité était comparable entre les deux sexes. Cette différence entre les deux sexes pourrait être expliquée d'une part, par l’âge des enfants compris entre 9 et 12 ans puisque, après un rebond pré-pubertaire, il y′a une augmentation de la masse grasse chez la fille alors que celle des garçons diminue [14, 15]. D'autres part, les garçons à cet âge seraient plus actifs [13]. Dans notre étude, la répartition des élèves selon leur statut pondéral par tranche d′âge avait montré que la fréquence de l′obésité était plus importante chez les enfants âgés de 11 à 12 ans, sans atteindre pour autant le seuil de la significativité. Ceci était également constaté par Gaha et al. [13].

D'autres facteurs peuvent influencer le profil pondéral des enfants, tel que leur poids à la naissance. Bedui et al. [12] en étudiant l'influence de la période prénatale sur la genèse de l'obésité chez l'enfant d’âge scolaire avait constaté qu'un poids à la naissance supérieur à 3.5 kg favorise le surpoids. Dans notre étude, la fréquence de surpoids (obésité incluse) était plus élevée dans le groupe d’élèves ayant un poids à la naissance plus que 4 kg ou inférieur que 2,5kg que les autres (10,7% vs 8,2%) sans que la différence soit statistiquement significative. De nombreuses études s'accordent pour montrer que la majorité des enfants obèses au début de leur vie ne le resteront pas [16]. Aussi, un rebond d'adiposité précoce, vers l’âge de trois ans, était retrouvé chez tous les enfants obèses. L’âge du rebond d'adiposité prédit l'adiposité à l’âge adulte [16]. Une durée courte (inférieure à 6 mois) d'allaitement et la diversification alimentaire précoce sont également pourvoyeurs de surcharge pondérale chez les enfants [12, 16]. Les enfants dont les parents sont obèses ont un risque important de devenir obèses [17, 18]. Whitaker et al. [16], en comparant les enfants dont les parents avaient un poids normal à ceux ayant des parents obèses, ces derniers avaient un risque d'obésité très augmenté (OR = 13,6). Dans notre étude il y avait une corrélation positive entre l'excès pondérale parentale et le risque d′obésité infantile (OR =2,62; p< 0,001). Cette association pourrait être expliquée par le déterminisme génétique et par le fait que les membres d'une même famille partagent le même style de vie, la même alimentation et le même niveau socioéconomique [19, 20]. Dans notre enquête, le haut niveau socio-économique avait émergé comme facteur favorisant le surpoids et l′obésité infantile. Ceci était également constaté dans d'autres études réalisées dans d'autres pays en voie de développement [16, 21, 22]. Paradoxalement, dans les pays développés, c'est le niveau socio-économique bas qui est généralement un facteur de risque de l'obésité chez les enfants [16, 17].

Dans une revue de la littérature, Newby [23] avait conclu qu'il y avait un manque de convergence des études sur le lien entre apports énergétiques et surpoids chez les enfants. Malgré ceci, le déséquilibre des apports en nutriment, était souvent évoqué comme risque de développer une obésité au cours de la croissance [14, 16]. Bellisle et al. [16] avaient rapporté que les enfants obèses mangeaient moins au petit déjeuner que les enfants de corpulence normale (15,7% vs 19% des apports énergétiques quotidiens), mais plus au diner (32,5% vs 28,7%). De même, Jouret et al. [16] avaient montré que les enfants obèses sautent deux à trois fois plus souvent le petit déjeuner. Le petit-déjeuner est souvent abandonné, ce qui peut entraîner des prises alimentaires incontrôlées en dehors des repas en raison de sensations de faim [16]. Dans notre étude, les enfants en surpoids avaient plus l′habitude de dépasser le petit déjeuner et de grignoter comparativement à ceux de corpulence normale, mais sans atteindre le seuil de la significativité. Par contre, nous avons trouvé une corrélation positive entre le surpoids et la prise de plus de 2 goûters par jour, aussi bien en analyse uni-variée que multi-variée.

Dans notre étude, les enfants en surpoids avaient plus l′habitude de dépasser le petit déjeuner ou de manger entre les repas et ils prenaient plus de 2 goûters par jour. Ce dernier résultat était confirmé même après ajustement par une analyse multi-variée. Malheureusement, il y′a aujourd′hui tendance à la détérioration des habitudes alimentaires chez les enfants en Tunisie comme dans tous les pays encerclant la méditerranée malgré que le régime méditerranéen ait gagné en popularité [12, 24]. Le bénéfice d'une activité physique régulière sur la composition corporelle et le développement de l'enfant était bien documenté. Les enfants actifs ont une masse grasse faible même si l'apport énergétique est élevé [16]. La fréquence de la surcharge pondérale était significativement plus élevée dans le groupe de jeunes qui n'avaient pas pratiqué de sport au lycée et ceux qui n’étaient pas affiliés à des associations sportives [13]. Dans notre étude, le fait de se rendre à l′école à pied, ou même à s′inscrire dans un club d′activité sportive n′a pas d′effet sur la prévalence de surpoids. L′absence d'associations entre l′activité physique et le surpoids pourrait être expliquée par les limites de notre questionnaire, puisque ce dernier ne permettait pas de mesurer objectivement les différents domaines de l′activité physique des enfants notamment les activités spontanées « d′agitation » que les études récentes n'ont pas cessé de montrer leur intérêt dans l′inégalité des individus vis à vis de la prise de poids [14].

Par contre, après l′analyse multi-variée, nous avons montré que la sédentarité (étudier à travers le nombre d′heures passées devant la télévision ou l′ordinateur et les jeux vidéo) était un facteur de risque de surpoids chez les enfants. Nos résultats corroborent celles de la plupart des études publiées à ce jour et qui montrent que ce n'est pas tant le lien entre l'activité physique et le surpoids qui semble être prépondérant mais plutôt celui entre sédentarité et surpoids. La diminution de l′activité physique entraînée par l′augmentation de la sédentarité serait plus importante que la diminution de la sédentarité engendrée par l′activité physique [25–27]. Le fait de réduire le temps passé devant la TV permettrait à l′enfant de bouger plus, manger mieux (moins de grignotages) et subir moins les influences publicitaires pour les aliments gras et sucrés [28, 29]. L'exploration et la prise en compte de la relation entre l′activité physique et la sédentarité permettront d’élaborer des stratégies de prévention et de cibler des messages de prévention [30].

## Conclusion

La prévalence de la surcharge pondérale et de l'obésité chez l'enfant est en augmentation galopante. Les chiffres apportés par les différentes études nous incites à tirer la sonnette d'alarme afin d'analyser les facteurs de risque de l'excès pondérale infantile dans le but de planifier et de mettre en place un programme de prévention entrepris très tôt dès l'enfance. Tout doit être mis en oeuvre pour améliorer ce profil de santé encourageant nos jeunes par la promotion d'une alimentation saine et l'instauration d'activité physique extrascolaire régulière. L'instauration des clubs de santé actifs dans l'ensemble de nos établissements serait d'une grande utilité dans ce contexte. Ces mesures de prévention ne devraient pas se limiter aux approches individuelles, mais devraient aussi inclure des mesures structurelles sur l'environnement social et physique de l'enfant.

## References

[2] Thibault H, Rolland-Cachera MF (2003). Prevention strategies of childhood obesity. Arch pediatr..

[3] Ben Slama F, Achour N (2007). L'obésité de l'enfant en Tunisie et dans le Monde. Institut National de la Santé Publique.

[4] Ben Slama F, Achour A, Belhadj O, Hsairi M, Oueslati M, Achour N (2002). Obésité et mode de vie dans une population d’écoliers de la région de l'Ariana. Tunisie Médical.

[5] Aounallah-Skhiri H, El Ati J, Traissac P, Ben Romdhane H, Eymard-Duvernay S, Delpeuch F (2012). Blood pressure and associated factors in a North African adolescent population. A national cross-sectional study in Tunisia. BMC Public Health.

[6] Harrabi I, Bouaouina M, Maatoug J, Gaha R, Ghannem H (2009). Prevalence of the metabolic syndrome among urban schoolchildren in Sousse, Tunisia. Int J Cardiol..

[7] Chiarelli F, Marcovecchio ML (2008). Insulin resistance and obesity in childhood. Eur J Endocrinol..

[8] Cole TJ, Bellizi MC, Flegal KM, Dietz WH (2000). Establishing a standard definition for child overweight and obesity worldwide: international survey. BMJ..

[9] Rolland-Cachera MF, Cole TJ, Sempe M, Tichet J, Rossignol C, Charraud A (1991). Body Mass Index variation: centiles from birth to 87 years. Euro J Clin Nutr..

[10] Molinari-Büchi B, Barth J, Janner M, Frey P (2010). Surcharge pondérale et obésité chez l'enfant: les acquis et les nouvelles tendances. Rev Med Suisse..

[11] Boukthir S, Essaddam L, Mazigh Mrad S, Ben Hassine L, Gannouni S, Nessib F (2011). Prevalence and risk factors of overweight and obesity in elementary schoolchildren in the metropolitan region of Tunis, Tunisia. La Tunisie Médicale..

[12] Bedoui A, Alouane L, Belhoula L (2004). Influence de la période périnatale sur la genèse de l'obésité chez l'enfant d’âge scolaire. XIIIéme Rencontre Scientifique de Nutrition, (résumé).

[13] Gaha R, Ghannem H, Harrabi A, Ben. Abdelaziz A, Lazreg F, Hadj Fredj A (2002). Etude de surcharge pondérale et de l'obésité dans une population d'enfants et d'adolescents scolarisés en milieu urbain à Sousse en Tunisie. Arch Pédiatr.

[14] Institut National de la Santé et de la Recherche Médicale (INSERM) (2000). Obésité: dépistage et prévention chez l'enfant. Paris édition Inserm.

[15] Jouret B, Tauber M Quels sont les enfants à risque de devenir des adultes obèses.

[16] Bouglé D, Vérine-Robine C, Duhamel JF (2001). Obésité de l'enfant: facteurs favorisants, prise en charge. Nutr Clin Métabol..

[17] Agence Nationale d'Accréditation et d'Evaluation en Santé (ANAES), service des recommandations professionnelles (2003). Prise en charge de l'obésité de l'enfant et de l'adolescent. http://www.anaes.fr.

[18] Magarey AM, Daniels LA, Boulton TJ, Cockington RA (2003). Predicting obesity in early adulthood from childhood and parental obesity. Int J Obes Relat Metab Disord..

[19] Speakman JR (2008). Thrifty genes for obesity, an attractive but flawed idea, and an alternative perspective: the ‘drifty gene’ hypothesis. Int J Obes (Lond).

[20] Hawkins SS, Law C (2006). A review of risk factors for overweight in preschool children: a policy perspective. Int J Pediatr Obes..

[21] Taleb S, Agli AN (2009). Obésité de l'enfant: rôle des facteurs socioéconomiques, obésité parentale, comportement alimentaire et activité physique, chez des enfants scolarisés dans une ville de l'Est algérien. Cahiers de Nutrition et de Diététique.

[22] Kelishadi R (2007). Childhood Overweight, Obesity, and the Metabolic Syndrome in Developing Countries. Epidemiol Rev..

[23] Newby PK (2007). Are dietary intakes and eating behaviors related to childhood obesity? A comprehensive review of the evidence. J Law Med Ethics..

[24] Frelut ML (2007). Obésité de l'enfant: regards et perspectives. Journal de pédiatrie et de puériculture..

[25] Rey-Lopez JP, Vicente-Rodriguez G, Biosca M, Moreno LA (2008). Sedentary behaviour and obesity development in children and adolescents. Nutr Metab Cardiovasc Dis..

[26] Prentice-Dunn H, Prentice-Dunn S (2012). Physical activity, sedentary behavior, and childhood obesity: a review of cross-sectional studies. Psychol Health Med..

[27] Duché P (2008). Activité physique et obésité infantile: dépistage, prévention et prise en charge. Sport et Science..

[28] Frelut ML, Peres G (2007). Activité physique et obésité de l'enfant: de sa responsabilité à son intérêt thérapeutique. Médecine thérapeutique/pédiatrie.

[29] DeMattia L, Lemont L, Meurer L (2007). Do interventions to limit sedentary behaviours change behaviour and reduce childhood obesity?. A critical review of the literature. Obes Rev..

[30] Simon C, Schweitzer B, Oujaa M, Wagner A, Arveiler D, Triby E (2008). Successful overweight prevention in adolescents by increasing physical activity: a 4-year randomized controlled intervention. Int J Obes (Lond).

